# Transcriptome-Wide Detection of Differentially Expressed Coding and Non-Coding Transcripts and Their Clinical Significance in Prostate Cancer

**DOI:** 10.1155/2012/541353

**Published:** 2012-08-16

**Authors:** Nicholas Erho, Christine Buerki, Timothy J. Triche, Elai Davicioni, Ismael A. Vergara

**Affiliations:** Department of Research and Development, GenomeDx Biosciences Inc., Vancouver, BC, Canada V6J 1J8

## Abstract

Prostate cancer is a clinically and biologically heterogeneous disease. Deregulation of splice variants has been shown to contribute significantly to this complexity. High-throughput technologies such as oligonucleotide microarrays allow for the detection of transcripts that play a role in disease progression in a transcriptome-wide level. In this study, we use a publicly available dataset of normal adjacent, primary tumor, and metastatic prostate cancer samples (GSE21034) to detect differentially expressed coding and non-coding transcripts between these disease states. To achieve this, we focus on transcript-specific probe selection regions, that is, those probe sets that correspond unambiguously to a single transcript. Based on this, we are able to pinpoint at the transcript-specific level transcripts that are differentially expressed throughout prostate cancer progression. We confirm previously reported cases and find novel transcripts for which no prior implication in prostate cancer progression has been made. Furthermore, we show that transcript-specific differential expression has unique prognostic potential and provides a clinically significant source of biomarker signatures for prostate cancer risk stratification. The results presented here serve as a catalog of differentially expressed transcript-specific markers throughout prostate cancer progression that can be used as basis for further development and translation into the clinic.

## 1. Introduction

Alternative splicing is a fundamental cellular process by which a multiexon gene generates different transcripts from the same primary sequence, thereby increasing functional diversity of the expressed genome. The central dogma of “one gene, one mRNA, and one protein” is outmoded as our understanding of the ubiquitous nature of gene splice variation; its complexity throughout normal development, cell differentiation, and in disease is better understood [1, 2].

The biological and clinical significance of differential expression of isoform variants is illustrated, for example, by the bcl-2 apoptotic gene family member bcl-x [3], for which the short (xS) and long (xL) variants are pro- and antiapoptotic, respectively. In prostate cancer, one of the most clinically relevant examples of differential expression of isoform variants has only recently been characterized for the androgen receptor (AR) [4–6]. While expression of the main isoform variant of AR is tightly coupled to sensitivity to antiandrogen therapy (AAT), the truncated v567 variant functions as a constitutively active, ligand-independent transcription factor that can support androgen-independent growth and progression of castrate-resistant metastatic prostate cancer. In general, due to the involvement of cancer-specific splice variants in very distinct molecular processes and their association with clinical outcome, they could be considered ideal candidates as diagnostic, prognostic, or predictive biomarkers [2]. Furthermore, the inclusion of splice variants might increase the specificity of previously identified “genes” as biomarkers and biomarker signatures. 

Recent advances in genome annotation and high-throughput technologies have led to the design of splicing-specific microarrays (e.g., exon, exon-junction, and tiling arrays) and RNA-sequencing (RNA-Seq), which allow transcriptome-wide expression profiling of coding and non-coding transcripts. While RNA-Seq is the technology of highest (i.e., single basepair) resolution and is especially powerful for the discovery of specific splice variants and novel transcripts, its utility in routine clinical testing remains to be proven. High-density microarrays, on the other hand, are already established tools in routine clinical testing (e.g., use of paraffin-embedded solid tumor specimens) [7, 8] and if analyzed correctly can provide a clear depiction of the transcriptome at exon-level resolution in both coding and non-coding genomic regions. In this study, we use Human Exon arrays to identify differentially expressed coding and non-coding transcripts involved in the progression continuum of prostate cancer by comparing normal prostate through metastatic disease tissue using transcript-specific probe selection regions (TS-PSR). Furthermore, we address the potential clinical significance of alternative splicing using TS-PSR biomarker signatures in comparison to established prognostic clinical variables and genes in prostate cancer.

## 2. Materials and Methods

### 2.1. Microarray and Clinical Data

The publically available genomic and clinical data was generated as part of the Memorial Sloan-Kettering Cancer Center (MSKCC) Prostate Oncogenome Project, previously reported by Taylor and colleagues [9]. The human exon array files for 131 primary prostate cancer tumors, 29 normal adjacent and 19 metastatic tissue specimens were downloaded from GEO at http://www.ncbi.nlm.nih.gov/geo/series GSE21034. The patient and specimen details for the primary and metastases tissues used in this study were reported elsewhere [9, 10].

### 2.2. Microarray Normalization and Summarization

The normalization and summarization of the 179 microarrays were done with the frozen Robust Multiarray Average (fRMA) algorithm using custom frozen vectors [11]. These custom vectors were created using the vector creation methods described in [12] including all MSKCC samples. Normalization was done by the quantile normalization method and summarization by the robust weighted average method, as implemented in fRMA. Gene-level expression values were obtained by summarizing the probe selection regions (or PSRs) using fRMA and the corresponding Affymetrix cluster annotation (http://www.affymetrix.com/).

### 2.3. Sample Subsets

The normalized and summarized data was partitioned into three groups. The first group contains the samples from primary localized prostate cancer tumor and normal adjacent samples (used for the normal versus primary comparison). The second group contains all the samples from metastatic tumors and all the localized prostate cancer specimens (used for the primary versus metastasis comparison). The third group corresponds to all samples from metastatic tumors and all the normal adjacent samples (used for the normal adjacent versus metastasis comparison).

### 2.4. Detection of Transcript-Specific PSRs in Human Exon Microarray Probe-Sets

Using the xmapcore R package [13], all PSRs overlapping with the exon of only one transcript were retrieved. This set of PSRs (hereafter called transcript-specific PSRs, or TS-PSRs) was further filtered in order to remove all those that correspond to a gene but such that (i) the gene has only one transcript or (ii) the gene has multiple transcripts, but only one can be tested in a transcript-specific manner (see Figure S1 in supplementary Material available online at doi:10.1155/2012/541353). In order to avoid complex regions, TS-PSRs overlapping with more than one gene (e.g., within the intron of another gene) on the same strand were filtered out from the analysis.

### 2.5. Feature Selection

PSRs annotated as “unreliable” by the xmapcore package [13] (one or more probes do not align uniquely to the genome) as well as those not defined as class 1 cross-hybridizing by Affymetrix were excluded from further analysis. Additionally, those PSRs that present median expression values below background level for all of the three tissue types (normal adjacent, primary tumor, and metastasis) were excluded from the analysis. The remaining TS-PSRs were subject to univariate analysis to discover those differentially expressed between the labeled groups (primary versus metastatic, normal adjacent versus primary, and normal adjacent versus metastatic). For this analysis, TS-PSRs were selected as differentially expressed if their adjusted false discovery rate (FDR) *t*-test *P* value is significant (<0.05) and the median fold difference (MFD) is greater than or equal to 1.2. The *t*-test was applied as implemented in the rowttests function of the genefilter package, http://www.bioconductor.org/packages/2.3/bioc/html/genefilter.html.

The multiple testing correction was applied using the p.adjust function of the stats package in R.

For any given transcript with two or more transcript-specific PSRs significantly differentially expressed the one with lowest *P*-value was chosen as representative of the differential expression of the transcript. 

### 2.6. Feature Evaluation and Model Building

A *k*-nearest-neighbor (KNN) model (*k* = 1, Euclidean distance) was trained on the normal adjacent and metastatic samples (*n* = 48) using only the top 100 *t*-test ranked features found to be differentially expressed between these two groups. 

### 2.7. Statistical Analysis

Biochemical recurrence endpoint is used as defined by the “BCR Event” column of the supplementary material provided by Taylor and colleagues [9]. Survival analysis for BCR was performed using the survfit function of the survival package in R. 

### 2.8. Annotation of Genes Known in Prostate Cancer

The list of differentially expressed genes was queried for previously reported association with prostate cancer by two means: (i) using E-utils PubMed Search; a gene is found associated with prostate cancer if it presents one or more hits in PubMed using the official gene symbol or any of the aliases in addition to the phrase “prostate cancer” found within the title or abstract and (ii) using a previously reported set of genes known to be differentially expressed in prostate cancer [14]. The list can be found in Supplementary Table 1.

Additionally, evidence for androgen regulation was pursued using the Androgen Responsive Gene Database, ARGDB [15]. The list of genes falling under this category can be found in Supplementary Table 2.

## 3. Results and Discussion

### 3.1. Detection of Transcript-Specific PSRs Using High-Density Microarrays

High-density Affymetrix human exon (“HuEx”) microarrays provide a unique platform to test the differential expression of the vast majority of exonic regions in the genome. Based on Ensembl v62 and xmapcore [13], there are 411,681 PSRs that fall within exons of protein-coding and non-coding (ncRNA) transcripts. Within this set, 123,521 PSRs (~10% of the total number of PSRs on the array) can be used for unequivocal testing of differential expression of alternatively spliced transcripts, as they overlap uniquely with the exon of only one splice variant. These PSRs, which we call transcript-specific PSRs (TS-PSRs), cover 49,302 transcripts corresponding to 34,599 genes.

In this study, we use the publicly available HuEx data set generated as part of the MSKCC Prostate Oncogenome Project [9] to explore transcript-specific differential expression through progression of prostate cancer from normal adjacent, primary tumor and metastatic tissues. In particular, we focus our analysis on the assessment of two or more distinct transcripts within a single gene or ncRNA to identify variants that may represent clinically and biologically relevant transcript-specific differential expression. This group of transcripts is the focus of the study since the TS-PSRs associated to them represent the most interesting cases in this technique and may lead to the discovery of novel diagnostic biomarkers and metastasis-specific druggable targets, such as those discovered for the AR isoform variants. The expression of TS-PSRs from genes for which only one transcript can be tested will either simply reflect the expression of that gene or will shed no light on the role of different transcripts within the same gene. Instead, genes with multiple TS-PSRs allow the detection of dominate variants and possibly a shift from one transcript variant to the other as the cancer progresses. Hence, the set of 123,521 TS-PSRs was further filtered in order to remove all those that correspond to a gene but such that (i) the gene has only one transcript (69,591 TS-PSRs; Supplementary Figure 1(a)), or (ii) the gene has multiple transcripts, but only one of these can be tested in a transcript-specific manner (14,927 TS-PSRs; Supplementary Figure 1(b)). This generates a final set of 39,003 TS-PSRs from 22,517 transcripts and 7,867 protein-coding and non-coding genes that are used as the basis of this analysis (Supplementary Figure 1(c)).

### 3.2. Differential Expression of Coding and Non-Coding Transcripts through Prostate Cancer Progression

**Figure 1:**
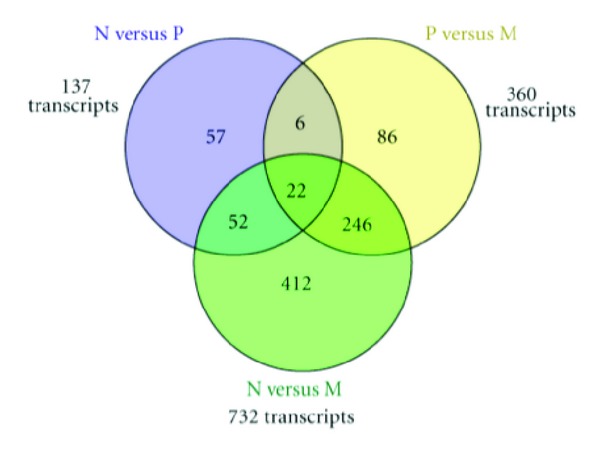
Venn diagram distribution of differentially expressed transcripts across pairwise comparison. N versus P: normal adjacent versus primary tumor comparison. P versus M: primary tumor versus metastatic sample comparison. N versus M: normal adjacent versus metastatic sample comparison.

Assessment of the defined set of TS-PSRs yielded 881 transcripts differentially expressed between any pairwise comparison of normal adjacent, primary tumor, and metastatic samples (see Section 2; Figure 1, Supplementary Table 2). These 881 transcripts correspond to 680 genes or ncRNAs with two or more transcripts differentially expressed at the same or different stages of cancer progression. Some of these are known prostate-associated protein-coding genes such as ACPP, TGM4, and STEAP2. While there are previous reports of transcript-specific differential expression for ACPP (*a.k.a.* PAP) and TGM4 [16, 17], to our knowledge this is the first report describing transcript-specific differential expression for STEAP2 (a.k.a. STAMP1), which is known to be differentially expressed in prostate cancer [18, 19]. 

Interestingly, 371 (42%) of the differentially expressed transcripts are non-coding. Inspection of their annotation reveals that they fall into several non-coding categories, the most frequent being “retained_intron” (*n* = 151) and “processed_transcript” (*n* = 186) (Supplementary Table 2). Additionally, most of the genes associated with these non-coding transcripts are coding, that is, the gene encodes at least one functional protein (although the specific isoform variant for that gene detected in this study does not). Examples of non-coding genes with differentially expressed transcripts found in this dataset include the lincRNAs PART1 (prostate androgen-regulated transcript 1) [20], MEG3 [21], the PVT1 oncogene, located in the 8q24 susceptibility region [22], and the testis-specific lincRNA TTTY10, which has been previously shown to be expressed in prostate [23]. Other ncRNAs include the small nucleolar RNA host gene 1 (SNHG1) which has been suggested as a useful biomarker for prostate cancer progression [24], as well as GAS5, located in the 1q25 risk loci [25]. Furthermore, three pseudogenes are found differentially expressed in this dataset including EEF1DP3, located in a region previously found to be a focal deletion in metastatic tumors [26] and the Y-linked pseudogene PRKY, which has been found expressed in prostate cancer cell lines [27]. 

In addition to the ncRNA genes, various coding genes present one or more non-coding transcripts differentially expressed. Many of these genes have been shown to be involved in prostate cancer and present evidence of androgen regulation (Table 1, Supplementary Table 2, see Section 2). These genes contain one or more non-coding transcripts differentially expressed in our analysis, including androgen receptor AR [4–6] and the fibroblast growth factor receptors FGFR1 and FGFR2 [28].

The set of non-coding transcripts in both coding and non-coding genes reported here add to the current stream of evidence showing that non-coding RNA molecules may play a significant role in cancer progression [10, 29].

Overall, of the 680 genes with one or more transcripts found differentially expressed, 281 have a previously reported association to prostate cancer (see Section 2). Still, the remaining 399 genes consist of 274 coding (31.1%) and 215 (24.4%) non-coding transcripts that are differentially expressed and originate from a gene with no previously described association with prostate cancer in the literature. These findings suggest that there remains much more to be discovered in prostate cancer and that the results presented here may represent important insights into biology with potential for clinical significance.

### 3.3. Genes with Multiple Transcripts Differentially Expressed through Prostate Cancer Progression

The majority of the 881 differentially expressed transcripts originate from the comparison between normal adjacent and metastatic samples, in agreement with previous analyses of differential expression in the MSKCC dataset [10]. As shown in Figure 1, 28 of the transcripts differentially expressed are found to represent a continuum of disease progression from normal adjacent through primary tumor and metastatic disease, with 22 of them across all three pairwise comparisons (Table 2, top). These 22 transcripts reflect instances of significant increase or decrease of expression through all stages in the same direction (*i.e.,* always upregulated or downregulated). The remaining 6 transcripts are found differentially expressed in the normal adjacent versus primary tumor as well as in the primary tumor versus metastatic sample comparison (but not in the normal adjacent versus metastatic samples comparison). These are a reflection of transcripts that play a role during the primary tumor stage of the disease (Table 2, bottom) In particular, within this set of 28 transcripts there are two AR-responsive genes, FGFR2 and NAMPT with two distinct transcripts each that are differentially expressed throughout the progression continuum. In the case of the FGFR2 gene, our observation of significant decrease in expression from normal adjacent to metastasis is in agreement with a previous study that shows downregulation of isoforms “b” and “c” associated with development of malignant prostate cancer [28]. In the case of NAMPT (a nicotinamide phosphoribosyltransferase), the two transcripts show highest expression in the primary tumor tissues compared to normal adjacent and metastasis; the rise in primary tumors compared to normal is in full agreement with previously reported elevation of expression during early prostate neoplasia for this gene [30].

**Figure 2:**
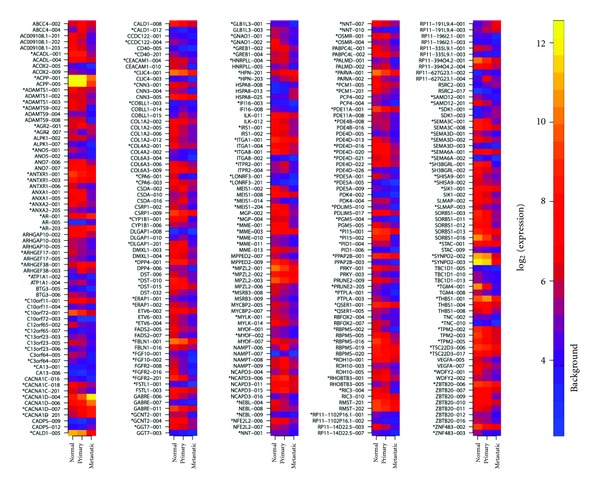
Heat map of genes with two or more transcripts differentially expressed across any pairwise comparison. Transcript names are provided as annotated in Ensembl. Heat map is colored according to median expression values for normal, primary and metastatic samples. “∗” indicates that the transcript is protein-coding. Background indicates the expression value considered as background level based on control probe sets on the HuEx array.

For FGFR2 and NAMPT, the transcripts happen to be differentially expressed in the same direction as the tumor progresses, suggesting that both transcripts are functioning in a cooperative manner. In order to determine if this is a general pattern of the transcripts analyzed here, all the genes for which at least two transcripts presented differential expression were inspected (Figure 2). Among the 140 genes for which such cases were found, there is a clear trend for groups of transcripts of the same gene to be expressed in the same “direction” of the tumor progression continuum. Two exceptions we found are genes CALD1 and AGR2. For both of these, differential expression of one of their transcripts in the progression from primary tumor to metastasis occurs in the opposite direction compared to the other transcripts. In the case of AGR2, transcript AGR2-001 is downregulated in metastasis compared to primary tumor, whereas AGR2-007 is upregulated. This observation is in agreement with previous reports for the pattern of expression for a short and long isoform of the same gene [31]. Even though the correspondence of the short and long isoforms to those annotated in Ensembl is not straightforward, alignment of the primers used by Bu and colleagues [31] shows overlapping of the short isoform with AGR2-001 and of the long isoform with AGR2-007, which agrees with the divergent expression patterns reported here (data not shown). In the case of CALD1, while transcript CALD1-012 is upregulated, CALD1-005 and CALD1-008 are downregulated in the progression from primary tumor to metastasis. A previous study on 15 prostate cancer samples shows that CALD1-005 is downregulated in metastatic samples compared to primary tumor, in agreement with our results [32].

### 3.4. Transcripts Level Resolution of Differential Expression on Fully Tested Genes

**Figure 3:**
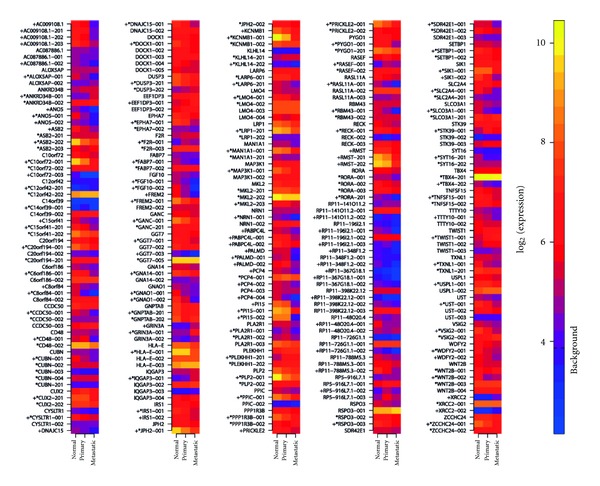
Heat map of genes for which all transcripts were assessed with one or more transcripts differentially expressed across any pairwise comparison. Transcript names are provided as annotated in Ensembl. Gene names are annotated based on their gene symbol. Heat map is colored according to median expression values for normal, primary and metastatic samples. “∗” indicates that the transcript is protein-coding. “+” indicates significant differential expression of a given transcript or gene. Background indicates the expression value considered as background level based on control probe sets on the HuEx array.

A particularly interesting group of genes for detection of differential expression is the one for which all annotated transcripts for a given gene can be tested individually (Supplementary Figure 2). Of the 7,867 genes for which one or more transcripts were assessed in this analysis, 1,041 genes are such that all of their transcripts have at least one TS-PSR (Supplementary Table 2). Of these, 92 genes have at least one of their transcripts differentially expressed in any pairwise comparison between normal adjacent, primary tumor, and metastatic samples. As depicted in Figure 3, the majority of the genes in this analysis have only one differentially expressed transcript. Examples of these are KCNMB1 and ASB2, two genes that have been previously reported to be differentially expressed in prostate cancer, but for which no observation at the transcript level has been made [33, 34]. In the case of KCNMB1, only transcript KCNMB1-001 of the two transcripts is found differentially expressed, whereas, for ASB2, only transcript ASB2-202 is found differentially expressed of the three transcripts annotated for this gene. Also, other protein-coding genes present differential expression of their non-coding transcripts only. One example of this is PCP4 (also known as PEP-19), a gene known to be expressed in prostate tissue [35]. Additionally, 15 of the 92 genes are non-coding. These genes include the testis specific lincRNA TTTY10 [23], the EEF1DP3 pseudogene in addition to others that have no previously reported association with prostate cancer, such as many of those derived from regions of the genome associated to RP11 and RP5 BAC clones.

In addition to the expression profile of each transcript for these 92 genes, Figure 3 shows the corresponding summarized gene-level expression profile for each gene. Of these, only 18 genes present differential expression that can be resolved at the gene level, clearly illustrating that summarization of expression to a “consensus gene” using these microarrays can result in a significant loss of information.

### 3.5. TS-PSRs Provide Clinically Significant Biomarker Signatures for Prostate Cancer Risk Stratification

**Figure 4:**
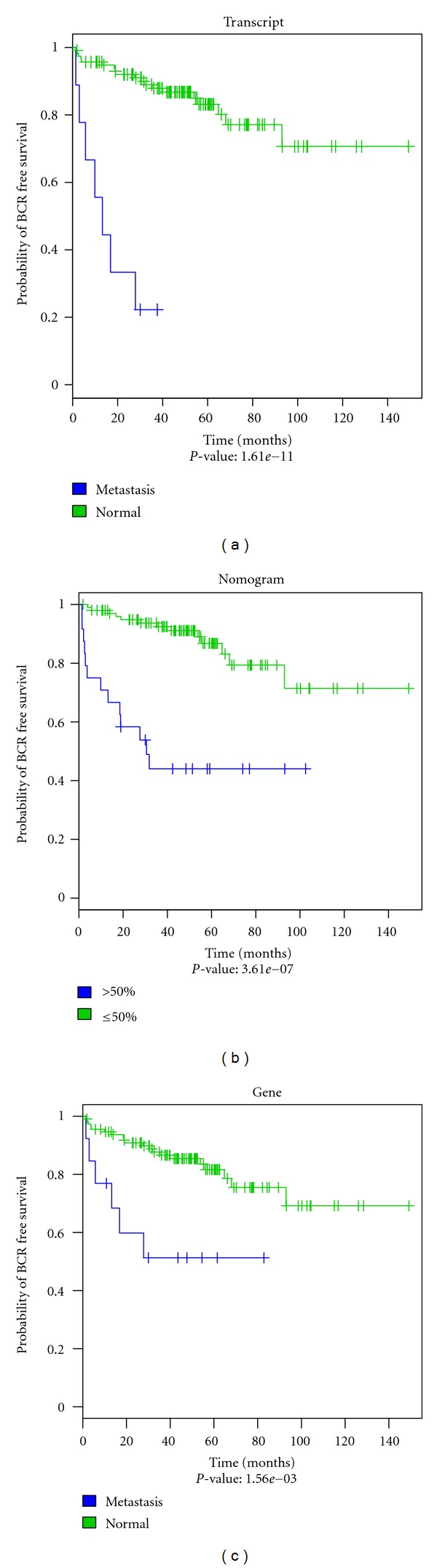
Kaplan Meier plots of primary tumor samples classified by KNN (“normal-like” versus “metastatic-like”) using the BCR endpoint. (a) Transcripts, (b) Kattan nomogram, and (c) genes. The blue curve indicates “metastasis-like” patients; the green curve indicates “normal-like” patients. For the nomogram a probability of greater than 50% for BCR was chosen to classify patients as “metastasis-like” or “normal-like.”

In order to assess the prognostic significance of the differentially expressed transcripts, the corresponding TS-PSRs were used to train a K-nearest neighbor (KNN) classifier on normal adjacent and metastatic samples. This KNN classifier was subsequently validated on the primary tumor subset, such that each primary tumor sample was classified as “normal-like” or “metastatic-like” based on its distance to the normal and metastatic groups. As shown in Figure 4, even though only a small fraction of the primary tumor patients were classified as “metastasis-like” due to the low risk nature of the cohort, the difference in the Kaplan-Meier (KM) curves for the two groups is statistically significant for the biochemical recurrence (BCR) endpoint and its performance is comparable to that of the Kattan nomogram for patient risk stratification [36]. Further assessment of coding and non-coding differentially expressed transcripts showed both sets to yield statistically significant differences in their KM curves (data not shown). The corresponding set of genes (i.e., nonisoform specific) also shows a statistically significant difference between the two risk groups in the KM curves albeit at a lower significance level, suggesting a loss of prognostic information when the data is summarized on the gene level. This is more apparent in a multivariable logistic regression analysis of the groups of transcripts and genes differentially expressed with adjustment for the Kattan nomogram (Table 3). While the isoform-specific transcripts dominate the multivariable model and remain highly significant (*P* < 0.005), the summarized genes do not dominate clinicopathological variables and as a result have a borderline significance for predicting BCR (*P* = 0.05). These results suggest that biomarker signatures based on specific transcript isoforms may offer unique prognostic information not captured by either summarized genes or clinicopathological variables and nomograms.

## 4. Conclusions

Transcriptome-wide detection of molecular markers for the development of better diagnostics and personalized medicine approaches have been facilitated by high-throughput technologies such as microarrays and more recently next-generation sequencing. Additionally, appreciation of the fact that most of the transcriptome is non-coding in both normal and cancer tissues [29] has significantly expanded the repertoire of available expressed biomarkers. In prostate cancer, a study by Chinnaiyan and colleagues using RNA-Seq highlights the existence of hundreds of non-coding RNAs that are differentially expressed between normal tissue and prostate tumor samples [37]. Additionally, a recent study by our group has shown that significant prognostic information is contained within the non-coding transcriptome of prostate cancer [10].

Gene expression profiling efforts in prostate cancer have not yet become mainstream. The argument against the use of gene-based biomarker signatures is that, despite numerous efforts, none have been shown to perform significantly better than established clinical variables and predictive models such as nomograms. Here, we demonstrate that improved predictive models can be obtained in prostate cancer by leveraging the complexity of transcript-specific isoforms.

In this study, we show that HuEx arrays are populated with thousands of probe selection regions (PSRs) that hybridize to a specific transcript (TS-PSRs). Given their unambiguous nature, these TS-PSRs become a useful and reliable tool to test the differential expression of individual transcripts across benign and cancerous tissues. Still, some of these TS-PSRs could be hybridizing to more than one transcript if additional transcripts of a given gene exist but have not been discovered yet and, hence, are missing from the genomic annotation. Even though we focus our analysis on a subset of TS-PSRs that correspond to 22,517 transcripts (from 7,867 genes) and that shed light on the behaviour of two or more transcripts within the same gene, the same approach can be generalized to 49,302 transcripts corresponding to 34,599 genes. The 881 transcripts found differentially expressed across normal adjacent, primary tumor, and metastatic prostate samples from the MSKCC Oncogenome Project [9] contained many cases previously reported in the literature to be involved in prostate cancer. Nevertheless, the disconnect between the names of variants for a given gene in the literature and the one provided in genome annotations such as Ensembl makes the comparison difficult to pursue in a case by case scenario and virtually impossible to automatize. Still, at the gene level it became evident that many genes associated with the 881 transcripts have been linked to prostate cancer but many of them have not been reported to play a role in prostate cancer progression. This is particularly true for non-coding variants of genes as well as non-coding genes such as lincRNAs and other unannotated genes, thus, adding to the stream of evidence that non-coding RNAs have significant potential for prognostic purposes.

In addition to genes presenting multiple transcripts differentially expressed as well as genes for which each individual transcript was probed, this study demonstrates that transcript-specific differential expression provides a clinically significant and unique source of biomarker signatures for prostate cancer risk stratification. We demonstrate that these biomarker signatures segregate patients into groups with significant differences in BCR-free survival and are significant prognostic factors for BCR prediction in multivariable analysis after adjusting for established prognostic factors such as the Kattan nomogram [36].

More datasets with associated clinical outcome are needed to further validate these findings. However, the results presented here serve as a catalog of differentially expressed transcript-specific markers throughout prostate cancer progression continuum that can be used as a basis for further exploration of disease biology and translation into clinical practice as novel diagnostics and therapeutics.

## Supplementary Material

Illustration of transcript-specific probe selection regions, list of genes found associated to prostate cancer, and list of transcripts found differentially expressed in this study.

## Figures and Tables

**Table 1:** Androgen-regulated genes known to play a role in prostate cancer with non-coding transcripts differentially expressed. All these genes present evidence of being androgen sensitive, based on ARGDB.

Gene	Transcript	Comparison
ABCC4	ABCC4-002	NvsP
	ABCC4-004	NvsP PvsM
ACADL	ACADL-001^∗^	PvsM NvsM
	ACADL-004	PvsM
ACPP	ACPP-001^∗^	PvsM
	ACPP-005	PvsM NvsM
ADAMTS1	ADAMTS1-001^∗^	NvsM
	ADAMTS1-002	NvsM
	ADAMTS1-003^∗^	NvsM
ANO7	ANO7-006	PvsM NvsM
	ANO7-007	PvsM NvsM
ANXA1	ANXA1-001	PvsM NvsM
	ANXA1-005	PvsM NvsM
AR	AR-001^∗^	NvsM
	AR-005	PvsM NvsM
	AR-203^∗^	NvsM
BNC2	BNC2-001	NvsM
BTG3	BTG3-005	PvsM NvsM
	BTG3-006	NvsM
CACNA1C	CACNA1C-016	NvsM
	CACNA1C-018^∗^	NvsM
	CACNA1C-201^∗^	NvsM
CACNA1D	CACNA1D-004^∗^	NvsM
	CACNA1D-006	PvsM NvsM
	CACNA1D-007^∗^	PvsM NvsM
	CACNA1D-201^∗^	PvsM NvsM
CALD1	CALD1-005^∗^	PvsM NvsM
	CALD1-008	PvsM NvsM
	CALD1-012^∗^	PvsM
CD40	CD40-005	NvsP NvsM
	CD40-201^∗^	NvsM
CD44	CD44-014	NvsM
CEACAM1	CEACAM1-004^∗^	PvsM NvsM
	CEACAM1-010	PvsM NvsM
COL1A2	COL1A2-002	NvsM
	COL1A2-005	NvsM
	COL1A2-006	NvsM
	COL1A2-012	NvsM
DPP4	DPP4-001^∗^	PvsM NvsM
	DPP4-006	PvsM NvsM
DST	DST-006	NvsM
	DST-010^∗^	PvsM NvsM
	DST-015^∗^	PvsM NvsM
	DST-032	NvsM
FBLN1	FBLN1-001^∗^	PvsM NvsM
	FBLN1-016	NvsM
FGFR1	FGFR1-005	NvsM
FGFR2	FGFR2-008	NvsP PvsM NvsM
	FGFR2-016^∗^	NvsP PvsM NvsM
	FGFR2-201^∗^	PvsM NvsM
GOLM1	GOLM1-008	NvsP
GSN	GSN-011	PvsM NvsM
HSPA8	HSPA8-008	PvsM
	HSPA8-013	PvsM NvsM
	HSPA8-025	PvsM
IFI16	IFI16-003^∗^	PvsM NvsM
	IFI16-008	PvsM NvsM
INSIG1	INSIG1-004	NvsM
IRS1	IRS1-001^∗^	PvsM NvsM
	IRS1-002	NvsM
KHDRBS3	KHDRBS3-003	PvsM
MAT2A	MAT2A-012	NvsP
MME	MME-001^∗^	PvsM NvsM
	MME-003^∗^	NvsM
	MME-010^∗^	NvsM
	MME-011^∗^	NvsM
	MME-013	NvsM
NAMPT	NAMPT-006	NvsP PvsM
	NAMPT-007	NvsP PvsM
	NAMPT-008	PvsM
	NAMPT-009	PvsM
NCAPD3	NCAPD3-004	PvsM
	NCAPD3-006^∗^	PvsM NvsM
	NCAPD3-011	PvsM
	NCAPD3-015	PvsM NvsM
	NCAPD3-016	PvsM
PALLD	PALLD-015	PvsM NvsM
PART1	PART1-001	PvsM
PBX1	PBX1-003	NvsM
PDE4B	PDE4B-008^∗^	PvsM
	PDE4B-016	PvsM
PDE4D	PDE4D-005	PvsM
	PDE4D-013	PvsM NvsM
	PDE4D-016^∗^	PvsM NvsM
	PDE4D-020^∗^	PvsM NvsM
	PDE4D-021^∗^	PvsM
	PDE4D-022	NvsM
	PDE4D-026	NvsM
PDLIM5	PDLIM5-010^∗^	PvsM
	PDLIM5-017	PvsM NvsM
PIK3R1	PIK3R1-008	NvsM
PPP2CB	PPP2CB-003	NvsM
RAN	RAN-006	PvsM NvsM
SEMA3C	SEMA3C-001^∗^	PvsM NvsM
	SEMA3C-008	PvsM NvsM
SVIL	SVIL-004	PvsM NvsM
TBC1D1	TBC1D1-005	NvsM
	TBC1D1-010	NvsM
	TBC1D1-013	NvsM
TGM4	TGM4-001^∗^	NvsP PvsM NvsM
	TGM4-008	NvsP NvsM
THBS1	THBS1-001^∗^	PvsM
	THBS1-004	PvsM
	THBS1-008	PvsM
TNC	TNC-002	PvsM NvsM
	TNC-010^∗^	PvsM NvsM
TPM2	TPM2-002^∗^	PvsM NvsM
	TPM2-003	PvsM NvsM
	TPM2-005^∗^	PvsM NvsM
TSC22D1	TSC22D1-004	PvsM
VCL	VCL-005	PvsM NvsM
VEGFA	VEGFA-005	NvsM
	VEGFA-007	NvsM
WSB1	WSB1-003	PvsM
XBP1	XBP1-005	PvsM NvsM
XRCC2	XRCC2-002	PvsM NvsM

^∗^indicates a protein-coding transcript. NvsP: normal adjacent versus primary tumor comparison. PvsM: primary tumor versus metastatic sample comparison. NvsM: normal adjacent versus metastatic sample comparison.

**Table 2:** Transcripts found differentially expressed across all pairwise comparisons (top) and across normal versus primary tumor and primary tumor versus metastatic samples comparisons (bottom).

Transcript	Mean fold difference		
	P versus N	M versus P	M versus N
ACOT11-001	0.79	0.77	0.61
AOX1-001	0.79	0.56	0.44
C19orf46-002	1.24	1.23	1.53
C8orf84-001	0.76	0.75	0.57
COCH-202	0.76	0.83	0.63
CTA-55I10.1-001	0.83	0.68	0.56
DMD-024	0.74	0.82	0.60
FGF10-002	0.83	0.64	0.53
FGFR2-008	0.76	0.79	0.60
FGFR2-016	0.74	0.67	0.49
GABRE-006	0.79	0.83	0.66
GNAL-001	0.82	0.69	0.57
GNAO1-002	0.78	0.75	0.58
HEATR8-006	0.80	0.80	0.64
ISL1-002	0.80	0.81	0.65
NR2F2-202	0.82	0.82	0.68
PCP4-004	0.81	0.72	0.58
PDE5A-005	0.74	0.79	0.59
PDZRN4-202	0.80	0.71	0.57
RSRC2-017	1.27	1.28	1.63
TGM4-001	0.68	0.62	0.42
TSPAN2-001	0.80	0.77	0.61

ABCC4-004	1.35	0.81	N.A.
ALK-001	1.24	0.83	N.A.
ATP1A1-002	1.23	0.71	N.A.
NAMPT-006	1.34	0.73	N.A.
NAMPT-007	1.75	0.57	N.A.
RP11-627G23.1-004	1.38	0.78	N.A.

N: Normal, P: Primary and M: Metastatic samples. N.A.: not applicable.

**Table 3:** Multivariable logistic regression analysis of transcripts and genes for prediction of BCR progression adjusted for Kattan nomogram.

Classifier	Transcripts			Genes		
	OR	OR CI (95%)	*P* value	OR	OR CI (95%)	*P* value
KNN positive**	13	[2.5–99]	<0.005	3.8	[1.0–14.3]	0.05
Nomogram^∗^	6.6	[2.3–20]	<0.001	7.9	[2.9–22.6]	<0.0001

**: metastatic-like. ^∗^: greater than 50% probability of BCR used as cut-off. OR: odds ratio. CI: confidence interval.
