# Supplementary figures and images for: Transcriptome-Wide Detection of Differentially Expressed Coding and Non-Coding Transcripts and Their Clinical Significance in Prostate Cancer
Source: J Oncol. 2012 Aug 16;2012:541353. doi: 10.1155/2012/541353 (PMC3431106; doi:10.1155/2012/541353)

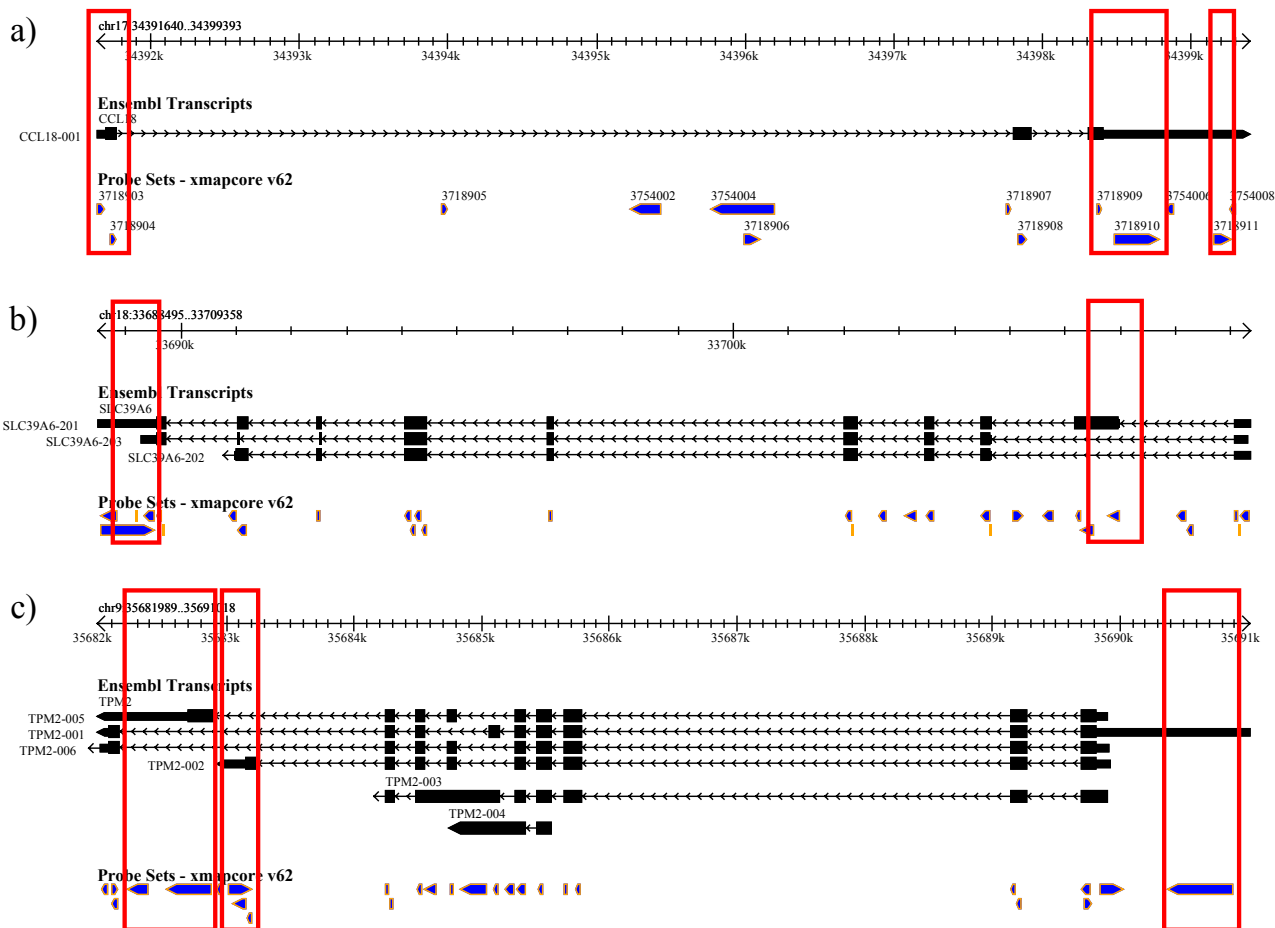

Supplement: Supplementary file 1 — Illustration of transcript-specific probe selection regions, list of genes found associated to prostate cancer, and list of transcripts found differentially expressed in this study. [file 541353.f1.pdf]

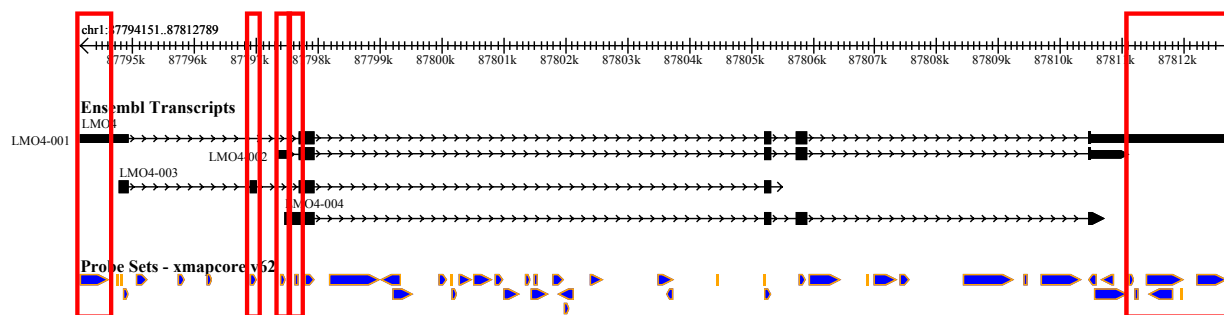

Supplement: Supplementary file 2 [file 541353.f2.pdf]
